# Effects of Different Extraction Methods on Vanilla Aroma

**DOI:** 10.3390/molecules27144593

**Published:** 2022-07-19

**Authors:** Chih-Hsin Yeh, Chia-Yi Chou, Chin-Sheng Wu, Lee-Ping Chu, Wei-Juan Huang, Hsin-Chun Chen

**Affiliations:** 1 Taoyuan District Agricultural Research and Extension Station, Council of Agriculture, Executive Yuan, Taoyuan 327, Taiwan; zeamays@tydais.gov.tw (C.-H.Y.); white981981@gmail.com (C.-Y.C.); 2 Department of Pharmacy, China Medical University Hospital, Taichung 404, Taiwan; m92189@mail.cmuh.org.tw; 3 Department of Orthopedics, China Medical University Hospital, Taichung 404, Taiwan; chu.leeping@gmail.com; 4 Department of Cosmeceutics, China Medical University, Taichung 406, Taiwan

**Keywords:** vanilla, GC-MS, volatile components, HS-SPME, SDE

## Abstract

To establish the analytic conditions for examining the aroma quality of vanilla pods, we compared different extraction methods and identified a suitable option. We utilized headspace solid-phase microextraction (HS-SPME), steam distillation (SD), simultaneous steam distillation (SDE) and alcoholic extraction combined with gas chromatography (GC) and gas chromatography–mass spectrometry (GC-MS) to identify volatile components of vanilla pods. A total of 84 volatile compounds were identified in this experiment, of which SDE could identify the most volatile compounds, with a total of 51 species, followed by HS-SPME, with a total of 28 species. Ten volatile compounds were identified by extraction with a minimum of 35% alcohol. HS-SPME extraction provided the highest total aroma peak areas, and the peak areas of aldehydes, furans, alcohols, monoterpenes and phenols compounds were several times higher than those of the other extraction methods. The results showed that the two technologies, SDE and HS-SPME, could be used together to facilitate analysis of vanilla pod aroma.

## 1. Introduction

Natural vanilla pods have a delicate and rich aroma that cannot be easily replicated and replaced by synthetic fragrances. As a result, with an increasing demand for vanilla pods, prices have rose, the market is in short supply, and there has been extensive news concerning the adulteration and blending of natural vanilla extracts [1]. Most foods release volatile organic compounds during storage or handling, which can be used as indicators of food quality or safety [2]. Thus, quick, stable and accurate extraction techniques are extremely important.

The techniques most commonly used to extract and analyze natural vanilla pods are alcoholic extraction, liquid–liquid extraction (LLE), and liquid–solid extraction (SLE) [3], as well as LLE with ultrasonic vibration, SDE and SPME, among others [4]. The ideal extraction technique must be able to extract the analyte quickly, easily, completely and inexpensively. Different extraction methods each have unique advantages but also have different usage limitations and disadvantages [5]. The extraction methods used in this experiment are introduced separately below.

Since vanilla pods are sold as alcoholic extracts in the international market [1], it is necessary to establish a suitable alcoholic extraction method for vanilla pods. According to the regulations of the U.S. Food and Drug Administration (FDA), the ethanol content of commercially available vanilla alcohol extracts should not be less than 35% (*v*/*v*).

Simultaneous steam distillation solvent extraction, a traditional extraction technique that is widely used to analyze volatile compounds [4], is a technique that combines solvent and steam distillation extraction, with better extraction efficiency than the former [6]. However, for many analyses, SDE is labor intensive, lacks sensitivity [7], requires large sample volumes, is time-consuming [8], and may raise concerns about solvent residues. In addition, under high-temperature extraction, some volatile compounds are easily hydrolyzed, thermally cracked or lost [7]. Cai et al. [4] also found that SDE is less sensitive to trace components. Nevertheless, the reproducibility of SDE is high, so SDE is the preferred choice for the quantitative analysis of volatiles.

Traditional methods of extracting volatile components are often time-consuming and prone to the loss or degradation of volatile components [9], in addition to low yields and the use of large amounts of solvents [1]. Therefore, modern scientists are devoted to finding extraction techniques that use low or even no solvent, thereby reducing the residual amount of harmful solvents in natural extracts [10]. SPME is a relatively new extraction technique [8] that is simpler than traditional methods [11], fast, solvent-free [7], environmentally friendly [3], does not thermally degrade or hydrolyze samples [4] and inexpensive [2]. Additional advantages without the need for time-consuming sample preparation are still needed [12], as well as strategies to reduce the harm caused by solvents to humans and the environment. Therefore, SPME has been applied in many fields, including agriculture, medicine [13], clinical testing, spice, food and environmental science [14]. This method has been demonstrated to rapidly extract volatile organic compounds (VOCs), and it is often used in GC and high-performance liquid chromatography (HPLC) to analyze the composition of complex volatile compounds in plants [9,11]. However, SPME also has disadvantages, which can lead to inaccurate quantification due to the adsorption competition of different volatile components. In addition, it has poor sensitivity and therefore cannot detect trace components [3].

Steam distillation extraction has been used to extract volatile compounds from medicinal plants [8] and is a traditional extraction technique used to separate essential oils from plants [15]. The principle is to use boiling water or steam to separate lower boiling volatile compounds from plant raw materials [16]. These water vapors and volatile oils are condensed through the condensing device and are called hydrosol and essential oil, respectively. The essential oil will float on the upper layer of the water layer (hydrosol) and can be effectively separated [15]. However, this extraction method is not only time-consuming and labor intensive [7] but also consumes a large number of samples. High-temperature extraction easily causes the loss of volatile compounds [17] or hydrolysis and oxidation of components [18].

The aim of this experiment was to explore, develop and verify different extraction methods and to find an analytical method suitable for extracting vanilla pods to establish the conditions for the aroma quality of vanilla pods, which can be used as a reference for the future development of the vanilla industry and aroma detection.

## 2. Results

### 2.1. Investigation of the Effect of Different Extraction Methods on the Aroma Components of Vanilla Pods

#### 2.1.1. SDE

In this experiment, pentane/ether (P/E) (1:1, *v*/*v*) was used for extraction. We chose a solvent with a low boiling point, which can be more easily removed to preserve the original aroma of vanilla pods [19]. Pérez-Silva et al. [20] compared the extraction of *V. planifolia* with pentane/dichloromethane (2:1, *v*/*v*), ether or pentane/ether (P/E) (1:1, *v*/*v*), and using P/E (1:1, *v*/*v*), the authors could extract a wide variety of compounds, potentially due to the difference in solvent polarity. According to Table 1, it can be observed that SDE could extract more carboxylic acids, aldehydes and phenols. Pérez-Silva et al. [20] extracted *V. planifolia* with P/E (1:1, *v*/*v*) and identified acids, phenols, alcohols, aldehydes, esters, hydrocarbons and ketones. The contents of acids and phenolic compounds were highest, among which the main aroma components were vanillin, vanillic acid and *p*-hydroxybenzaldehyde.

Although the types of components were similar to those identified in this experiment, vanillic acid was not identified in this experiment, probably because the gas chromatography column used by the author was polar (DB-WAX), and herein we used a nonpolar column (DB-1). Table 2 shows that SDE could extract palmitic acid and other larger-molecule components. Cai et al. [4] believed that SDE could be used to extract compounds with larger molecular weights and lower volatility, such as palmitic acid, compared with HS-SPME. Bajer et al. [21] considered SDE to be a more suitable extraction technique for analyzing volatile components with high retention indices (RIs). The present study showed that the volatile components with higher RIs were only identified by the SDE extraction method, which was consistent with previous studies.

#### 2.1.2. HS-SPME

A total of 28 volatile compounds were identified by HS-SPME extraction of vanilla pod samples (Table 2). The samples contained 6 aldehydes, 6 phenols, 5 alcohols, 3 esters, 2 ketones, 2 hydrocarbons, 2 sesquiterpenes, 1 furan and 1 monoterpene. The total peak area with HS-SPME was the largest and the total peak area of aldehydes was more than 5 times greater than that obtained with the other extraction methods (Table 1). In addition, the total peak areas of furans, alcohols and phenols were also higher than those obtained with the other extraction methods. The main components of vanilla pods analyzed by HS-SPME were phenol, 1-octen-3-ol, 2-pentylfuran, 1-octanol, guaiacol and vanillin. Yeh et al. [22] used HS-SPME to analyze *V. planifolia* produced in Taiwan and detected a variety of monoterpenes and sesquiterpenes. Among them, limonene, α-copaene and α-muurolene were also identified in the experiment, which can offer vanilla citrus, lemon and wood aromas. Hassan et al. [12] analyzed *V. planifolia* using HS-SPME and showed that shikimate derivatives accounted for the majority of *V. planifolia*, and vanillin was the most abundant component. In addition, volatile compounds, such as benzaldehyde, *p*-anisaldehyde, *p*-hydroxybenzaldehyde, benzyl alcohol, *p*-cresol, guaiacol, creosol and *p*-anisyl alcohol, were all shikimic acid derivatives. In this experiment, such compounds accounted for approximately 92% of the components, among which vanillin was the most abundant, followed by guaiacol. Although guaiacol was abundant, it is generally considered to have a negative effect on vanilla pod aroma [23], and with increasing guaiacol content, the vanillin content tends to decrease [24]. 

Compared with other extraction methods, HS-SPME extracted more monoterpenes and sesquiterpenes. Although the total peak area of HS-SPME was highest, no carboxylic acid compounds were identified, and the types of compounds were lower than those obtained with SDE. Kraujalytė et al. [25] found that HS-SPME was more suitable for compounds with low volatility due to the lower extraction temperature. Therefore, this extraction method was consistent with previous studies and is suitable for simple and rapid detection of sample components [4].

#### 2.1.3. SD

A total of 25 volatile compounds were identified using SD extraction of vanilla pod samples (Table 2). The samples contained 11 aldehydes, 5 ketones, 4 esters, 3 alcohols, 1 phenol and 1 hydrocarbon. In this experiment, SD could not extract important aroma components, such as *p*-hydroxybenzaldehyde and vanillin, from vanilla pods, possibly because *p*-hydroxybenzaldehyde [26] and vanillin are only slightly soluble in water (1 g/100 mL) [1]. Additionally, the aqueous layer of SD extract lacks compounds, such as *p*-hydroxybenzaldehyde and vanillin. Despite the absence of vanillin, the total peak areas of aldehydes still accounted for 68% of the extract (as shown in Table 1), which might be related to the greater polarity of aldehydes. From Table 3, it can be observed that a large amount of furfural appeared in the extract. Cai et al. [4] speculated that this phenomenon was caused by the hydrolysis and pyrolysis of the compounds during the extraction process.

#### 2.1.4. Alcoholic Extraction

In this experiment, 35, 75 and 95% alcohol were used to extract vanilla pods, and 10, 14 and 19 volatile compounds were identified, which consisted of only aldehydes, esters, carboxylic acids, alcohols, ketones and phenols. According to Table 2, the contents of guaiacol, *p*-hydroxybenzaldehyde and vanillin extracted from vanilla pod with 35% alcohol were lower than those in the other two ethanolic extracts. Moreover, esters and carboxylic acids were only identified in the 75% and 95% ethanolic extractions but not in the 35% ethanolic extraction. However, only the 35% ethanolic extracts contained vanillyl alcohol. Hernández-Fernández et al. [37] used GC–MS to compare the differences between 35% ethanolic extraction (1:10, *v*/*v*) and supercritical carbon dioxide extraction of *V. planifolia*. They found that the vanilla pod ethanolic extract contained six compounds, guaiacol, *p*-vinylguaiacol, vanillin, *p*-hydroxybenzaldehyde, vanillyl alcohol and vanillic acid. Excluding vanillic acid, the other five compounds were detected in the 35% ethanolic extract in this experiment. Sostaric et al. [9] extracted *V. planifolia* with 35% alcohol, and the extraction ratio was consistent with this experiment (1:5, *v*/*v*). Additionally, they used GC–MS to compare differences between the *V. planifolia* ethanolic extract and synthetic flavor. The authors found that natural vanillin extracts contain high amounts of vanillin and long carbon-chain esters that are not found in synthetic flavors such as ethyl nonanoate and ethyl decanoate. Synthetic fragrances contain ethyl vanillin that are lacking in natural vanilla extracts. Comparing three kinds of vanilla pod extracts with different alcohol concentrations, it can be observed that the higher the alcohol concentration, the more volatile components are extracted and the greater are the total peak areas. At present, commercial vanilla alcohol extracts are mostly extracted with 35% (*v*/*v*) alcohol [37], potentially because higher alcohol concentrations will alter the vanilla aroma of the extract. However, consumer acceptance is not high. Hernández-Fernández et al. [37] believed that alcohol extraction has some disadvantages, such as high concentration of organic residues, longer extraction time, and a larger dosage required for use as a spice.

### 2.2. Quantitative Analysis of Vanilla Pods

In this experiment, SDE was used to quantitatively analyze vanilla pod samples, and a total of 51 volatile compounds were identified (Table 3) using the method that identified the most compounds among all evaluated extraction methods. It contained 9 aldehydes, 10 carboxylic acids, 9 phenols, 7 esters, 6 hydrocarbons, 4 alcohols, 2 ketones, 2 sesquiterpenes, 1 furan and 1 monoterpene, revealing that the content of vanillin was highest, followed by guaiacol. Januszewska et al. [38] found that the main volatile components of vanillin pods from different origins were vanillin and guaiacol. Among them, vanillin has sweet and creamy aromas and is an important aroma component of vanilla pods [39]. Zhang and Mueller [19] quantified the volatile components of *V. planifolia* extracts by GC–MS and identified *p*-hydroxybenzaldehyde, (*E*)-methyl cinnamate, benzyl alcohol, phenol, *p*-cresol, 1-octanol, 2-phenylethanol, benzoic acid, octanoic acid, creosol, methyl salicylate, anisaldehyde, nonanoic acid, anisyl alcohol, isovanillin and other volatile compounds, and these compounds were also identified in this experiment. Among them, the content of guaiacol, a minor component, was 105.00 mg/kg, which was similar to the quantification results (101.58 mg/kg). In addition, guaiacol, creosol and phenol endow *V. planifolia* with strong phenolic, woody and smoky flavors [40].

### 2.3. Comparison of Different Extraction Methods

**Figure 1:**
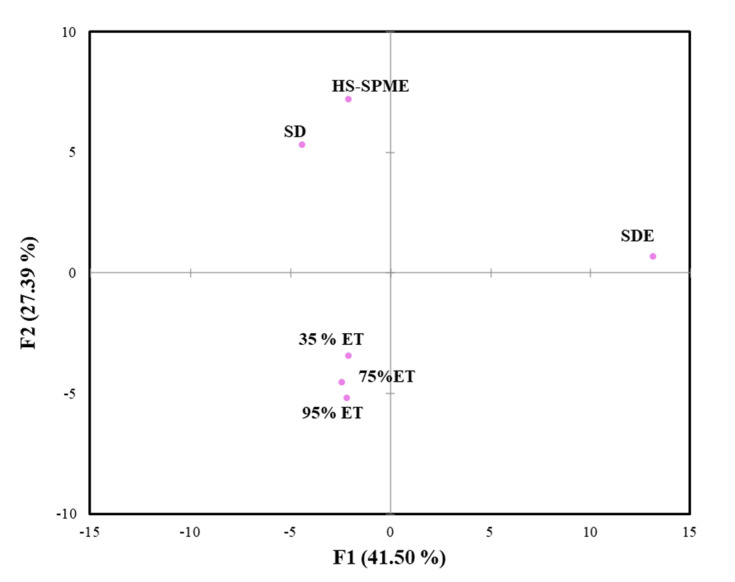
Principal component analysis diagram (PCA) of vanilla pods with different extraction methods. ●: Samples (ET: ethanolic extract).

Figure 1 shows a principal components analysis (PCA) diagram of different extraction methods, from which it can be observed that the different methods can be divided into 3 groups. The three ethanolic extracts with different concentrations were close to the same group on the PCA diagram, which indicated that the composition of ethanolic extracts with different concentrations were similar. Table 2 also shows that the volatile components extracted with the three different concentrations of alcohol were mainly composed of aldehydes, alcohols, ketones and phenols, which can be compared with the PCA results. SDE could extract a wide variety of volatile components. In addition, in contrast to the other extraction methods, the proportion of aldehydes was highest, while SDE had the highest content of acid components, and no carboxylic acid compounds were identified in SD and HS-SPME (Table 2). Therefore, SDE was the farthest from other extraction methods on the PCA diagram, and it can be speculated that the volatile components extracted with SDE were the most different from other extraction methods.

Vanillin is the main component of natural vanilla pods, so the content of vanillin is extremely important for vanilla extracts [1]. In SD extracts, vanillin cannot be detected, so this method is preliminarily considered unsuitable for analysis of vanillin. Although most commercially available vanilla pods are sold in the form of ethanolic extraction, the number of components and total peak areas identified by ethanolic extraction in this study were the lowest. Zheng et al. [41] compared the extraction of Syringa flowers with different solvents, and they also found that the efficiency of ethanolic extraction was poor. Based on the results of this experiment, it was found that SDE could extract more volatile components, but the total peak areas of HS-SPME were more than twice as large as those obtained with SDE. In addition, this study showed that only HS-SPME and SDE could extract monoterpenes and sesquiterpenes. Kung et al. [31] used SDE and HS-SPME to analyze the volatile compounds from *Platostoma palustre* and found that SDE could extract more volatile compounds and sesquiterpenes. However, HS-SPME could extract more monoterpenes than SDE. In this study, the monoterpene total peak areas of HS-SPME were higher while the sesquiterpene total peak areas were lower than those determined with SDE, which was similar to the results of a previous study. For many assays, SDE lacks the sensitivity and convenience required for experiments, and HS-SPME can make up for these shortcomings. Cai et al. [4] believed that the reproducibility of SDE was better than that of HS-SPME, so if quantitative analysis is needed, SDE is the best extraction method. In addition, SDE can extract more components. However, it is less sensitive to trace components. Reineccius [42] pointed out that no method will accurately reflect the aroma components actually present in a food or their proportions. Therefore, it is recommended to use SDE and SPME complementary to analyze more complete vanilla aroma components. 

## 3. Materials and Methods

### 3.1. Plant Materials

In this experiment, top bourbon vanilla beans (*V. planifolia*) with similar length and weight (about 17 cm and 4 g) which had been cultivated and cured in Sava, Madagascar, and were purchased from MR. Vanilla Beans commercial source in Taiwan.

### 3.2. Extraction Method

#### 3.2.1. HS-SPME

The 65 μm PDMS/DVB adsorption fibers used in this experiment were purchased from Supelco, Bellefonte, PA, USA. The experimental procedure has been described by Yeh et al. [22]; 8–10 vanilla pods were cut in half, and 1 g of vanilla seeds were scraped and placed into a 4 mL cylindrical glass bottle with a Teflon rubber pad. It was then heated in a 50 °C water bath and extracted with a 65 μm PDMS/DVB adsorption fiber for 40 min. After the extraction was completed, GC and GC–MS desorption were applied for 20 min for analysis in splitless mode. The above process was repeated 3 times.

#### 3.2.2. SDE

A total of 20 g vanilla pods were cut into approximately 0.2 cm wide pieces and placed in a 5 L three-necked round bottom flask. Then, 500 g water and 1.00 g internal standard (0.5 mg/g cyclohexyl acetate) were added, and a Likens-Nickerson (L-N) device was connected. Fifty milliliters of *n*-pentane/diethyl ether at a ratio of 1:1 (*v*/*v*) was added to the bottom of the L-N device, placed in a pear-shaped bottle as a solvent end, and then placed in a water bath at 40–50 °C. The other end was connected to a 5 L three-neck round-bottom flask filled with 4 L of water as a heat source for steam distillation, and the sample end was heated to 100 °C. After extraction for 2 h, the solvent extract in the pear-shaped bottle was collected, dehydrated with anhydrous sodium sulfate and filtered with No. 1–125 mm qualitative filter paper. Then, a distillation column device (40 °C, 1 h, 100 cm glass column) was used to remove excess solvent and collect the concentrated volatile compound extract. GC syringes were used to collect 1 μL, and GC and GC–MS analyses were performed by direct injection. The split ratio was 1:100. The above process was repeated 3 times.

#### 3.2.3. SD

Twenty grams of vanilla pods were cut into approximately 0.2 cm wide pieces and placed into a 5 L three-necked round-bottom flask. Then, 500 g of water was added, the other end and connected to a 5 L three-necked round-bottomed flask, and 4 L of water was placed in the flask for steam distillation. The sample end was heated to 100 °C. After 2 h, the extract was collected, and 10 g was placed in a 15 mL cylindrical glass bottle with a Teflon rubber pad. Then, the samples were extracted with 65 μm PDMS/DVB adsorption fibers of HS-SPME for 40 min at room temperature. After the extraction was completed, GC and GC–MS desorption were used for 20 min for analysis in splitless mode. The above process was repeated 3 times.

#### 3.2.4. Alcoholic Extraction

Two grams of vanilla pods were cut into approximately 0.2 cm wide pieces, and 20 g of 95, 75 and 35% alcohol was added. After extraction with an ultrasonic shaker for 30 min, the mixture was shaken by hand for 1 min and filtered with No. 1–125 mm qualitative filter paper. The filtrate was collected for later use. Twenty grams of 95, 75 and 35% alcohol was added to the vanilla pod sample again and the above extraction method repeated. The two extracts were mixed and filtered with anhydrous sodium sulfate, and the extract was injected into the capillary using a 3 mL disposable syringe to remove excess solvent and concentrated. One microliter of the extract was collected with GC syringes and analyzed by GC and GC–MS by direct injection with a split ratio of 1:10. Each of the above alcohol concentrations was repeated 3 times.

### 3.3. Internal Standard (IS) Preparation

Standard compound of cyclohexyl acetate was purchased from Sigma-Aldrich (St. Louis, MO, USA). cyclohexyl acetate (0.5 g) was diluted to 10 g with 95% alcohol and then serially diluted to 0.5 mg/g.

### 3.4. GC/GC-MS Instrument Analysis

#### 3.4.1. GC

The instrumental conditions refer to Yeh et al. [22]. The instrument used in this study was an Agilent Model 7890 GC (Santa Clara, CA, USA), and the separation column was a DB-1 (60 m × 0.25 mm i.d.) from Agilent, which is a nonpolar column. The carrier gas was nitrogen (N_2_) delivered at a flow rate of 1 mL/min. The injection port temperature was set to 250 °C. The detector was a flame ionization detector (FID), and the detector temperature was 300 °C. The oven temperature was maintained at 40 °C for 1 min, then raised to 150 °C at 5 °C/min, held for 1 min, raised to 200 °C at 10 °C/min, and then maintained at this temperature for 21 min.

#### 3.4.2. GC-MS

A Model 5977A quadrupole mass spectrometer (Mass Selective Detector, MSD) from Agilent (CA, USA.) was used. The ion source temperature of the MSD was 230 °C, and the quadrupole temperature was 150 °C. The GC was an Agilent Model 7890B. The operating conditions for the GC and the use of column were the same as those described for GC, changing only the carrier gas to helium (He). The mass spectral data measured by the instrument were compared with the mass spectral library of Wiley 7N.

### 3.5. Quantitative Calculation of the IS Method

The IS method is a relatively accurate quantitative method in instrumental analysis, and its calculation formula is as follows:

$$\mathrm{Sample}\;\mathrm{concentration}\;(\mathrm{mg}/\mathrm{kg})=\frac{{(A}_{x}{)(C}_{\mathrm{is}})}{{(A}_{\mathrm{is}}{)(W}_{s})}\;\times \;1000$$

where *A_x_* = The peak area of the compounds in the sample,

*A_is_* = the peak area of IS,

*C_is_* = the amount of IS added (mg), and

*W_s_* = the sample weight (g).

### 3.6. Statistical Analysis

In this study, principal component analysis (PCA) was performed using XLSTAT2014 (Addinsoft, New York, NY, USA). The data were subjected to one-way analysis of variance, with Tukey’s multiple range method used to identify significant differences of *p* < 0.05 with GraphPad Prism 5 (GraphPad Software, San Diego, CA, USA).

## 4. Conclusions

From the PCA chart, it can be observed that the different extraction methods could be divided into 3 groups. Among them, the three different concentrations of alcohol were extracted from the same group, and the composition was similar. They were mainly composed of aldehydes, alcohols, ketones and phenols. However, Alcohol extraction at 35% resulted in the fewest extraction components. In this experiment, SD extraction could not detect vanillin, so this method is not suitable for analysis of vanilla pods. SDE could extract a variety of volatile compounds, while HS-SPME did not extract the most components but could extract more aroma total peak areas. The result suggested that the HS-SPME and SDE are both powerful analytic tool for the determination of the volatile compounds in vanilla. Therefore, HS-SPME is recommended for the preliminary identification of vanilla aroma. Otherwise, SPME and SDE can complement each other for vanilla aroma analysis. 

## Figures and Tables

**Table 1:** Total peak areas of the chemical groups of vanilla pods using different extraction methods.

Chemical Groups	Peak Areas ^1^					
	SDE	SD	HS-SPME	SE		
				35%	75%	95%
aldehydes	3593.07	3383.06	21,546.27	989.18	2096.52	2266.22
esters	396.12	319.49	174.65	-	22.45	22.25
furans	16.11	- ^2^	289.08	-	-	-
monoterpenes	13.19	-	24.58	-	-	-
sesquiterpenes	68.26	-	55.22	-	-	-
carboxylic acids	3882.94	-	-	-	12.68	39.62
alcohols	164.39	425.67	934.03	14.36	33.76	46.43
ketones	137.94	627.84	204.12	210.39	110.28	365.43
phenols	2306.55	175.39	6104.42	48.92	118.94	117.61
hydrocarbons	779.67	28.73	30.10	-	-	-
total	11,391.62	4960.18	29,362.47	1262.85	2394.63	2857.56

^1^ Each value is the mean of three replication. ^2^ undetectable.

**Table 2:** Analysis of the volatile components of vanilla pods after different extractions methods.

Compounds ^1^	RI ^2^	Peak Areas ^3^					
		SDE	SE			SD	HS-SPME
			35% Ethanol	75% Ethanol	95% Ethanol		
ethyl acetate	601	24.34 ± 9.04	- ^4^	-	-	-	-
3-methylbutanal	627	-	-	-	-	873.24 ± 113.12	-
3-methylpentanal	740	-	-	-	-	306.10 ± 145.40	-
hexanal	772	-	-	-	-	846.53 ± 16.78	-
1,3-butanediol	777	-	-	-	-	-	117.55 ± 18.55
furfural	790	41.05 ± 9.58c	-	-	20.32 ± 3.09c	159.07 ± 6.22a	127.28 ± 15.98b
furfuryl alcohol	844	-	27.62 ± 2.73a	33.76 ± 14.29a	37.67 ± 10.91a	-	-
heptanal	874	-	-	-	-	128.62 ± 16.41	-
5-methyl-2(5H)-furanone	886	-	-	24.69 ± 11.47	-	-	-
5-methylfurfural	921	-	-	7.44 ± 0.70b	10.19 ± 1.22a	-	-
benzaldehyde	922	25.94 ± 5.46c	-	-	-	246.10 ± 6.85a	84.20 ± 13.60b
phenol	947	151.63 ± 31.97b	11.52 ± 2.05c	25.65 ± 2.86c	14.92 ± 1.97c	-	428.03 ± 52.91a
1-octen-3-one	948	-	-	-	-	61.90 ± 4.79	-
2-octanone	954	-	-	-	-	203.83 ± 28.07	-
1-octen-3-ol	955	-	-	-	-	47.87 ± 11.27b	279.51 ± 53.18a
2-pentylfuran	968	16.11 ± 4.99b	-	-	-	-	289.08 ± 59.24a
octanal	971	-	-	-	-	265.90 ± 59.40	-
hexanoic acid	975	60.46 ± 37.02	-	-	-	-	-
benzyl alcohol	992	22.11 ± 6.29b	-	-	-	-	114.58 ± 14.82a
phenylacetaldehyde	996	39.00 ± 8.68a	-	-	-	17.18 ± 3.86b	-
3-octen-2-one	999	15.71 ± 4.24c	-	-	-	221.40 ± 19.36a	108.05 ± 15.40b
limonene	1010	13.19 ± 4.09a	-	-	-	-	24.58 ± 5.86a
furaneol	1011	-	-	-	9.41 ± 4.18	-	-
*p*-cresol	1037	50.37 ± 13.50b	4.75 ± 0.52c	14.09 ± 5.72c	10.87 ± 2.95c	-	144.94 ± 20.72a
1-octanol	1041	117.30 ± 30.42b	-	-	-	321.86 ± 37.59a	380.32 ± 40.93a
guaiacol	1052	1747.13 ± 405.33b	11.34 ± 0.97c	24.61 ± 2.28c	24.41 ± 5.69c	175.39 ± 11.37c	5315.06 ± 911.24a
2-nonanone	1059	-	-	-	-	85.18 ± 7.09a	96.07 ± 11.61a
nonanal	1070	31.47 ± 10.74b	-	-	-	333.49 ± 41.50a	88.34 ± 12.20b
2-phenylethanol	1073	17.35 ± 4.16b	-	-	-	-	42.07 ± 1.94a
2-(1- methylethylidene)cyclohexanone	1088	-	-	-	-	55.54 ± 6.39	-
methyl octanoate	1091	-	-	-	-	35.06 ± 3.14	-
1,2-dimethoxybenzene	1096	-	-	-	-	-	15.84 ± 1.59
2,3-dihydro-3,5-dihydroxy-6-methyl-4H-pyran-4-one	1102	-	81.02 ± 18.00a	85.59 ± 17.35a	75.80 ± 25.06a	-	-
benzoic acid	1122	-	-	12.68 ± 3.87	-	-	-
3,5-dimethylphenol	1131	8.25 ± 3.90	-	-	-	-	-
octanoic acid	1144	194.34 ± 50.37	-	-	-	-	-
2-nonenal	1151					148.27 ± 75.52	-
creosol	1157	86.53 ± 22.09b	-	28.13 ± 0.79c	-	-	189.44 ± 18.06a
methyl salicylate	1163	26.58 ± 8.42c	-	-	-	231.96 ± 19.16a	109.37 ± 10.96b
safranal	1170	-	-	-	-	58.55 ± 32.52a	19.77 ± 4.98a
5-hydroxymaltol	1170	-	129.37 ± 69.97a	-	280.22 ± 66.22a	-	-
5-hydroxymethylfurfural	1172	-	-	324.78 ± 52.36	-	-	-
3-phenyl-1-propanol	1193	7.63 ± 2.96	-	-	-	-	-
methyl nonanoate	1195	12.63 ± 7.97b	-	-	-	41.40 ± 2.70a	31.79 ± 2.42a
dodecane	1200	-	-	-	-	-	19.40 ± 1.32
anisaldehyde	1210	17.43 ± 6.08	-	-	-	-	-
chavicol	1218	11.44 ± 1.09	-	-	-	-	-
cinnamaldehyde	1229	14.81 ± 4.42	-	-	-	-	-
anisyl alcohol	1243	-	-	-	8.76 ± 1.81	-	-
nonanoic acid	1255	1014.60 ± 250.70a	-	-	29.60 ± 1.39b	-	-
(*E*)-methyl cinnamate	1268	24.46 ± 7.74a	-	-	-	11.07 ± 3.90b	33.49 ± 1.57a
*p*-vinylguaiacol	1280	167.46 ± 39.17a	21.30 ± 14.47b	26.47 ± 9.23b	27.03 ± 4.18b	-	-
2,4-decadienal	1284	69.98 ± 17.33	-	-	-	-	-
*p*-hydroxybenzaldehyde	1313	35.10 ± 12.64c	92.54 ± 23.72b	160.39 ± 7.12a	195.96 ± 25.85a	-	24.78 ± 1.63c
methyl anisate	1337	29.85 ± 5.05	-	-	-	-	-
decanoic acid	1341	120.36 ± 4.45	-	-	-	-	-
(*Z*)-methyl cinnamate	1349	208.71 ± 34.92	-	-	-	-	-
vanillin	1358	3318.29 ± 552.20b	896.65 ± 243.99b	1603.90 ± 114.67b	2026.60 ± 409.18b	-	21,216.89 ± 7078.54a
α-copaene	1380	24.90 ± 6.47b	-	-	-	-	44.72 ± 3.48a
tetradecane	1400	-	-	-	-	-	10.69 ± 1.15
2,6-dimethylnaphthalene	1405	-	-	-	-	28.73 ± 8.69	-
methylparaben	1410	-	-	22.45 ± 1.94a	22.25 ± 1.93a	-	-
veratraldehyde	1424	-	-	-	-	-	9.78 ± 0.53
vanillyl alcohol	1425	-	14.36 ± 3.35	-	-	-	-
undecanoic acid	1434	59.82 ± 24.10	-	-	-	-	-
1-dodecanol	1450	-	-	-	-	55.94 ± 11.95	-
2,4-di-tert-butylphenol	1484	37.65 ± 14.08a	-	-	10.36 ± 0.59b	-	-
butylated hydroxytoluene	1491	46.08 ± 13.19a	-	-	30.02 ± 10.05ab	-	11.11 ± 1.04b
α-muurolene	1496	-	-	-	-	-	10.50 ± 4.32
lauric acid	1535	271.98 ± 19.08	-	-	-	-	-
hexadecane	1600	28.29 ± 17.89	-	-	-	-	-
syringaldehyde	1613	-	-	-	13.15 ± 2.62	-	-
tridecanoic acid	1629	43.26 ± 9.38	-	-	-	-	-
cadalene	1660	43.36 ± 14.21	-	-	-	-	-
heptadecane	1700	54.54 ± 15.57	-	-	-	-	-
myristic acid	1731	363.16 ± 59.98a	-	-	10.02 ± 0.77b	-	-
1-octadecene	1757	56.26 ± 36.74	-	-	-	-	-
octadecane	1800	71.81 ± 25.42	-	-	-	-	-
6,10,14-trimethylpentadecan-2-one	1817	122.22 ± 29.90	-	-	-	-	-
pentadecanoic acid	1823	268.47 ± 38.16	-	-	-	-	-
nonadecane	1900	382.03 ± 24.96	-	-	-	-	-
methyl palmitate	1926	69.55 ± 33.23	-	-	-	-	-
palmitic acid	1962	1486.50 ± 159.94	-	-	-	-	-
eicosane	2000	132.20 ± 72.64	-	-	-	-	-

^1^ Tentatively identification of components based on GC-MS library (Wiley 7n). ^2^ Retention indices, using paraffin (C_5_–C_25_) as references. ^3^ Total peak areas from GC-FID, values are means ± SD of triplicates. Different letters within the same line denote significant difference in Tukey’s multiple test (*p* < 0.05). ^4^ undetectable.

**Table 3:** SDE quantifies the volatile components of vanilla pods.

Compounds ^1^	RI ^2^	RI ^3^	Concentration (mg/kg) ^4^	References
ethyl acetate	603	601	1.39 ± 0.26	[19]
furfural	799	790	2.39 ± 0.33	[22]
benzaldehyde	931	922	1.52 ± 0.24	[27,28]
phenol	949	947	8.86 ± 1.37	[29]
2-pentylfuran	975	968	0.92 ± 0.11	[22]
hexanoic acid	955	975	3.13 ± 1.34	[30]
benzyl alcohol	1011	992	1.27 ± 0.09	[28]
phenylacetaldehyde	1002	996	2.28 ± 0.39	[31]
3-octen-2-one	1015	999	0.90 ± 0.07	[19]
limonene	1017	1010	0.75 ± 0.03	[31,32]
*p*-cresol	1043	1037	2.90 ± 0.28	[22]
1-octanol	1048	1041	6.76 ± 0.69	[31]
guaiacol	1056	1052	101.58 ± 13.92	[22]
nonanal	1074	1070	1.79 ± 0.19	[22]
2-phenylethanol	1080	1073	1.01 ± 0.12	[22]
3,5-dimethylphenol	1139	1131	0.40 ± 0.21	[33,34]
octanoic acid	1150	1144	11.21 ± 1.14	[19]
creosol	1161	1157	5.01 ± 0.66	[22]
methyl salicylate	1166	1163	1.51 ± 0.13	[22,31]
3-phenyl-1-propanol	1201	1193	0.42 ± 0.03	[19]
methyl nonanoate	1205	1195	0.69 ± 0.32	[19]
anisaldehyde	1212	1210	0.98 ± 0.04	[22]
chavicol	1223	1218	0.54 ± 0.02	[19]
cinnamaldehyde	1239	1229	0.85 ± 0.09	[19]
nonanoic acid	1247	1255	58.74 ± 7.10	[19]
(*E*)-methyl cinnamate	1281	1268	1.39 ± 0.04	[33,34]
*p*-vinylguaiacol	1280	1280	9.91 ± 2.42	[22]
2,4-decadienal	1288	1284	4.10 ± 0.81	[33,34]
*p*-hydroxybenzaldehyde	1315	1313	1.98 ± 0.22	[19,22]
methyl anisate	1336	1337	1.41 ± 0.15	[33,34]
decanoic acid	1344	1341	7.43 ± 2.91	[19]
(*Z*)-methyl cinnamate	1356	1349	12.45 ± 3.14	[30]
vanillin	1354	1358	196.36 ± 40.91	[28]
α-copaene	1373	1380	1.46 ± 0.33	[35,36]
undecanoic acid	1445	1434	3.41 ± 0.74	[33,34]
2,4-di-tert-butylphenol	1494	1484	2.10 ± 0.14	[33,34]
butylated hydroxytoluene	1488	1491	2.64 ± 0.23	[33,34]
lauric acid	1566	1535	16.59 ± 5.68	[19]
hexadecane	1600	1600	1.54 ± 0.60	[19]
tridecanoic acid	1645	1629	2.57 ± 0.65	[33,34]
cadalene	1653	1660	2.51 ± 0.52	[19]
heptadecane	1700	1700	3.12 ± 0.19	[19]
myristic acid	1739	1731	21.56 ± 4.89	[33,34]
1-octadecene	1788	1757	3.49 ± 2.35	[33,34]
octadecane	1800	1800	4.03 ± 0.31	[19]
6,10,14-trimethylpentadecan-2-one	1817	1817	7.08 ± 0.86	[33,34]
pentadecanoic acid	1823	1823	16.00 ± 3.85	[33,34]
nonadecane	1900	1900	24.01 ± 11.20	[19]
methyl palmitate	1909	1926	3.81 ± 0.99	[19]
palmitic acid	1968	1962	90.12 ± 28.34	[33,34]
eicosane	2000	2000	7.04 ± 2.43	[28]

^1^ Tentatively identification of components based on GC-MS library (Wiley 7n). ^2^ literature retention indices obtain from [19,22,27,28,29,30,31,32,33,34,35,36] and reference were checked for all on DB-1. ^3^ Retention indices, using paraffin (C_5_–C_25_) as references. ^4^ Total concentration from GC-FID, values are means ± SD of triplicates.

## Data Availability

Data sharing not applicable. No new data were created or analyzed in this study. Data sharing is not applicable to this article.

## Sample Availability

Samples of the compounds are not available from the authors.
